# Tear nanoDSF Denaturation Profile Is Predictive of Glaucoma

**DOI:** 10.3390/ijms24087132

**Published:** 2023-04-12

**Authors:** Viktoriia E. Baksheeva, Veronika V. Tiulina, Elena N. Iomdina, Sergey Yu. Petrov, Olga M. Filippova, Nina Yu. Kushnarevich, Elena A. Suleiman, Rémi Eyraud, François Devred, Marina V. Serebryakova, Natalia G. Shebardina, Dmitry V. Chistyakov, Ivan I. Senin, Vladimir A. Mitkevich, Philipp O. Tsvetkov, Evgeni Yu. Zernii

**Affiliations:** 1 Belozersky Institute of Physico-Chemical Biology, Lomonosov Moscow State University, 1-40 Leninskye Gory, 119992 Moscow, Russia; 2 Institut Neurophysiopathol, INP, Faculté des Sciences Médicales et Paramédicales, Aix Marseille Univ, CNRS, 13005 Marseille, France; 3 Helmholtz National Medical Research Center of Eye Diseases, 105062 Moscow, Russia; 4 Université Jean Monnet Saint-Etienne, CNRS, Institut d Optique Graduate School, Laboratoire Hubert Curien UMR 5516, 42023 Saint-Etienne, France; 5 Engelhardt Institute of Molecular Biology, Russian Academy of Sciences, 119991 Moscow, Russia

**Keywords:** biomarker, diagnostic, glaucoma, POAG, tear fluid, tear proteins, lysozyme C, lipocalin-1, IgA, serum albumin, lactotransferrin, fatty acids, iron, thermal denaturation, nanoDSF

## Abstract

Primary open-angle glaucoma (POAG) is a frequent blindness-causing neurodegenerative disorder characterized by optic nerve and retinal ganglion cell damage most commonly due to a chronic increase in intraocular pressure. The preservation of visual function in patients critically depends on the timeliness of detection and treatment of the disease, which is challenging due to its asymptomatic course at early stages and lack of objective diagnostic approaches. Recent studies revealed that the pathophysiology of glaucoma includes complex metabolomic and proteomic alterations in the eye liquids, including tear fluid (TF). Although TF can be collected by a non-invasive procedure and may serve as a source of the appropriate biomarkers, its multi-omics analysis is technically sophisticated and unsuitable for clinical practice. In this study, we tested a novel concept of glaucoma diagnostics based on the rapid high-performance analysis of the TF proteome by differential scanning fluorimetry (nanoDSF). An examination of the thermal denaturation of TF proteins in a cohort of 311 ophthalmic patients revealed typical profiles, with two peaks exhibiting characteristic shifts in POAG. Clustering of the profiles according to peaks maxima allowed us to identify glaucoma in 70% of cases, while the employment of artificial intelligence (machine learning) algorithms reduced the amount of false-positive diagnoses to 13.5%. The POAG-associated alterations in the core TF proteins included an increase in the concentration of serum albumin, accompanied by a decrease in lysozyme C, lipocalin-1, and lactotransferrin contents. Unexpectedly, these changes were not the only factor affecting the observed denaturation profile shifts, which considerably depended on the presence of low-molecular-weight ligands of tear proteins, such as fatty acids and iron. Overall, we recognized the TF denaturation profile as a novel biomarker of glaucoma, which integrates proteomic, lipidomic, and metallomic alterations in tears, and monitoring of which could be adapted for rapid non-invasive screening of the disease in a clinical setting.

## 1. Introduction

Glaucoma is a neurodegenerative ocular disorder (optical neuropathy) representing the leading cause of blindness globally. It encompasses a heterogeneous group of conditions affecting mainly the optic nerve and retinal ganglion cells (RGCs) and is conventionally classified into primary (idiopathic, congenital) and secondary (pigment dispersion syndrome, pseudoexfoliation syndrome, iatrogenic glaucoma, drug-induced glaucoma, etc.) forms, which can be further divided into open-angle and angle-closure variants, depending on the morphology of the anterior chamber angle [1]. Primary open-angle glaucoma (POAG) is the most common form of the disease, the prevalence of which in the European population was estimated to be 2.6% and showed a dramatic increase with age [1,2]. The main risk factor for POAG is an elevation of intraocular pressure (IOP) up to 20–30 mmHg due to impeded aqueous humor (AH) derange via the anterior chamber angle (through the trabecular meshwork/Schlemm canal) and/or the uveoscleral pathway. Chronic ocular hypertension leads to cupping of the optic nerve head in lamina cribrosa and may trigger RGC apoptosis both directly, via mechanical stress and interrupting axonal transport, and indirectly, through zinc and/or glutamate toxicity, vascular dysregulation, retinal ischemia, and oxidative stress, among other mechanisms [3,4,5,6]. The early stages of POAG are often asymptomatic since RGC deaths and defects of the visual field accumulate first on the retinal periphery. However, without treatment, the progression of glaucomatous neurodegeneration ends with irreversible loss of central vision and, in some cases, full blindness [3]. The preservation of visual function in POAG patients critically depends on the timely application of adequate IOP-lowering therapy via medicinal and surgical interventions, and this requires a prompt and reliable establishing diagnosis of the disease.

Currently, the gold standard for POAG diagnostics includes tonometry (increased IOP), biomicroscopic examination of the optic nerve (increased cup-to-disc ratio and other signs), perimetry (visual field defects), and optical coherence tomography (thinning of retinal nerve fiber layer) [1,7]. However, these tests have several limitations. Indeed, IOP measurement does not provide any information about the rate of glaucomatous neurodegeneration, the noticeable changes in the visual field manifest only in developed stages of the disease, whereas interpretation in the last two techniques is subjective, depends on the anatomical features of a patient, and requires conventional standards, which are currently absent [7]. The key to reliable diagnostics can be the determination of robust molecular biomarkers exhibiting clear correlations with POAG development. Being a multifactorial disease, glaucoma affects most of the eye tissues and liquids, which can serve as sources of such biomarkers. Recent advances in mass-spectrometric technologies triggered their search among the metabolome, lipidome, and proteome of AH, tear fluid (TF), and plasma of patients [8,9,10,11]. The most relevant source for glaucoma biomarkers is AH, as it directly accumulates proteins and metabolites that were secreted by cells and tissues affected by the disease, thereby representing its biochemical fingerprint [7,9]. However, AH can be collected only by an invasive procedure, which is feasible in patients with advanced glaucoma during antiglaucoma surgery but hardly suitable for nominally healthy individuals undergoing routine medical screenings.

TF represents a more convenient subject for analysis due to the simplicity and non-invasiveness of collection, and TF biomarkers of glaucoma have gained increasing attention over the last few years [12,13]. It was hypothesized that tears may become enriched in glaucoma-related molecules directly from the AH via scleral percolation in the uveoscleral pathway [14]. Accordingly, there are correlations between the molecular contents of AH and TF in some species, including humans [9,15]. Growing evidence indicated that glaucomatous degeneration is indeed associated with characteristic alterations in TF components, such as soluble metabolites or lipids [9,16,17]. Furthermore, a number of promising biomarkers of glaucoma were revealed among TF proteins [12]. Some investigators suggest using single protein biomarkers of POAG, such as BDNF, matrix metallopeptidases, or cytokines [18,19,20]. The detection of these proteins in TF can be facilitated by novel techniques, including lateral flow assay and the use of microfluidic paper-based devices [13]. However, most studies using mass-spectrometric approaches indicate that POAG is associated with complex proteomic changes in TF [17,21,22]. Even though examination of the whole TF proteome may help in establishing POAG diagnosis, the total amount of proteins to be analyzed in this case is quite large (>1500 [23]), and the required mass-spectrometry study and data processing seem too cumbersome to be used in clinical practice.

Previously, an original approach allowing for the simultaneous comparison of large groups of proteins, such as proteomes of plasma, was proposed for diagnostic purposes. The approach is based on the non-conventional employment of differential scanning calorimetry (DSC) or differential scanning fluorimetry (nanoDSF) complemented by artificial intelligence (AI) analysis [24,25,26,27,28]. It was demonstrated that registration of thermal denaturation profiles of total plasma with these techniques can be used for non-invasive diagnostics of glioblastoma or other cancers and apparently is sensitive enough to detect their molecular subtypes [28,29]. In the current study, we trialed a similar concept of glaucoma detection based on the monitoring of thermal denaturation of TF proteins followed by an AI analysis of obtained denaturation profiles. To this end, we used a rapid high-performance nanoDSF instrument, requiring small amounts of sample and performing measurements in 3 min. Using a wide cohort of 311 ophthalmic patients, we demonstrated the overall high potential of such a diagnostic approach. Furthermore, we identified characteristic POAG-associated alterations in core TF proteins underlying the changing of tear denaturation properties in the course of the disease. We believe that the proposed approach could be adapted for rapid non-invasive screening for glaucoma in a clinical setting.

## 2. Results

### 2.1. Patient Characteristics

The study involved a total of 311 participants (Table 1). The POAG group included 82 patients with a median age of 69 years (from 43 to 83 years), containing approximately equal proportions of individuals with mild (Stage I), moderate (Stage II), and advanced (Stage III) forms of the disease. All participants presented characteristic signs of POAG, including chronically elevated IOP, an increased mean cup-to-disc ratio, and visual field abnormalities. The control group included 171 patients with refractive anomalies but without a history of glaucoma or dry eye. Since, in the elderly, individuals with such characteristics are rare and may have latent glaucoma, we recruited a broad-age control cohort (8–77 years; median age 32 years) while taking the age differences into account in subsequent analysis. Most of the POAG patients received long-term instillations of IOP-lowering drugs, such as β-blockers, inhibitors of carbonic anhydrase, and prostaglandin analogs, which were also considered during their classification. To trial the feasibility of the invented approach for differential diagnostics, we also recruited a group of 58 patients with peripheral retinal degenerations (PRDs), which are common lesions, such as lattice degeneration. Being mechanistically different from glaucoma, PRD seems to be associated with developmental anomalies, and its prevalence is increased in myopic patients. Although most PRD forms are clinically insignificant, they are associated with an increased risk of rhegmatogenous retinal detachments, an important cause of severe visual impairments [30]. PRD patients did not exhibit statistically significant age differences as compared to control individuals, while all three groups were similar in gender proportion.

### 2.2. TF Profiling Using nanoDSF

**Figure 1:**
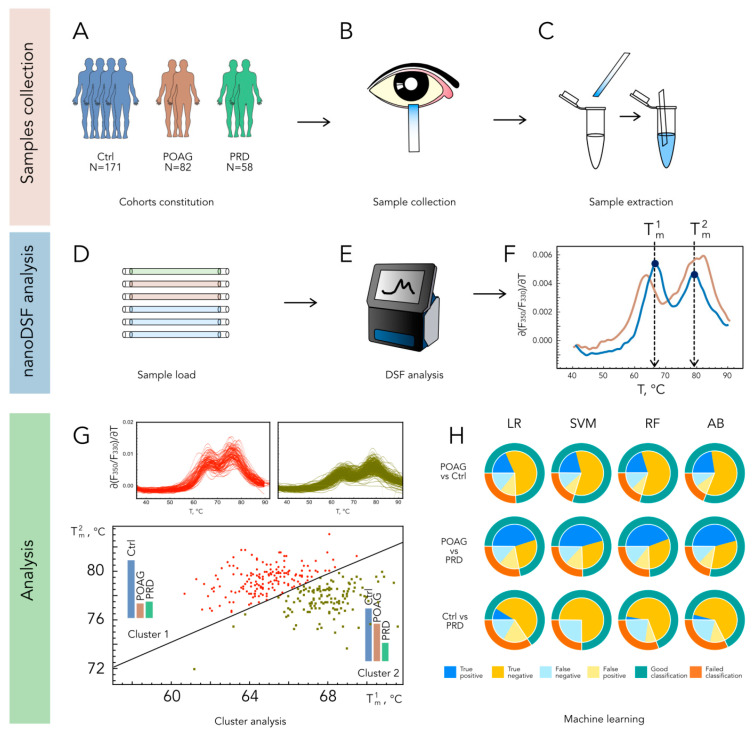
A workflow designed for the identification of POAG patients based on TFDP. Tear samples are collected using Schirmer tear strips in a cohort of individuals with refraction abnormalities (Ctrl), POAG, or PRD (**A**–**C**) and analyzed by nanoDSF yielding typical profiles with two peaks exhibiting characteristic shifts in POAG ((**D**–**F**); normal and POAG-type TFDPs are indicated in blue and brown, respectively). The classification of patients is accomplished using k-means two-parameter clustering of the profiles according to peaks maxima ((**G**); TFDPs belonging to clusters 1 and 2 are indicated in red and willow green, respectively), or AI analysis (**H**), where nanoDSF outputs are tested as input data for machine learning algorithms Logistic Regression (LR), Support Vector Machine (SVM), Random Forest (RF), and Adaptive Boosting (AB).

TF samples were collected from all trial populations by using gauged Schirmer’s test paper strips (Figure 1A,B). The procedure took 5 min and did not require anesthesia or tear stimulation. To achieve a reliable analysis of TF, we employed an original procedure for its recovery from the Schirmer’s strips, without using specific extraction solutions (see Section 4), thus allowing us to maintain the content of the tear proteins and their complexes. At the end of the recovery procedure, the TF sample remained at the bottom of a microcentrifuge tube, from which it could be conveniently aspirated by capillaries of the nanoDSF instrument (Figure 1C–E). The denaturation profiles of TF were registered in the range from 35 to 95 °C, using Tycho NT.6. Each TF denaturation profile (TFDP) consists of six curves: temperature dependence of fluorescence intensity at two wavelengths, 330 nm and 350 nm; the ratio of fluorescence intensities at 350 nm and 330 nm; and their derivatives. For visualization purposes and further cluster analysis, we used only the temperature dependence of ∂(F_350_/ F_330_)/∂T (Figure 1, F and top panels of G). The respective dependencies registered for samples collected from the patients with POAG, PRD, and control groups exhibited a characteristic shape, with two major peaks with maxima at around 65 °C (T^1^_m_) and 78 °C (T^2^_m_) (Figure 1F).

### 2.3. Analysis of TF Denaturation Profiles

The primary classification of the patients was performed by considering only T1m and T2m values. The k-means clustering based on these parameters yielded two distinct populations (Cluster 1 and Cluster 2), characterized by average T^1^_m_/T^2^_m_ values of 67.9/77.7 °C and 64.6/79.3 °C, respectively (Figure 1G and Supplementary Table S1). Notably, Cluster 2 contained the majority of POAG patients (72%), whereas the PRD and control groups fell into both clusters in almost equal proportions (Table 2 and Supplementary Table S1). A comparison of POAG groups corresponding to each cluster revealed no significant differences in age, gender, or treatment regimens (Table 2).

The main limitation of the two-parameter clustering was the high number of false-positive identifications, i.e., when healthy individuals were recognized as POAG patients. To increase the accuracy of the identification, we analyzed the obtained TFDPs by using an AI approach. Four machine learning algorithms were employed (LR, SVM, RF, and AdaBoost), which performed well in the classification of cancer patients based on the nanoDSF profiles of plasma proteins [27]. In this case, the input data included temperature dependencies of F_330_, F_350_, and F_350_/F_330_, as well as their first derivatives. In the pair “POAG versus control”, all algorithms gave a similar accuracy of 75–80% (Figure 1H). The most beneficial was the AdaBoost algorithm (accuracy of 81.4%), for which the correct identification of POAG patients was achieved in 70% of cases, whereas the amount of false-positive controls was relatively low (13.5%, Table 2). The identified groups of true-positive and false-negative POAG patients did not differ in median age, gender, or treatment, suggesting that the provided classification relied on biochemical alterations in TF related to the disease. Notably, the groups of true positives determined via k-means two-parameter clustering and the AI/AdaBoost approach contained 80% of the same POAG patients. Similar to two-parameter clustering, the AI analysis failed to reliably discriminate PRD patients from the control group (Table 2). Accordingly, the data analysis in the pair “PRD versus POAG” allowed for the significant discrimination of these diseases with an accuracy of 75%.

Based on these findings, we can suggest that TF profiling by nanoDSF powered by the described classification methods can be regarded as the basis for the development of a rapid non-invasive approach for POAG diagnostics.

### 2.4. Assessment of POAG-Related Alterations in TF Proteins

**Figure 2:**
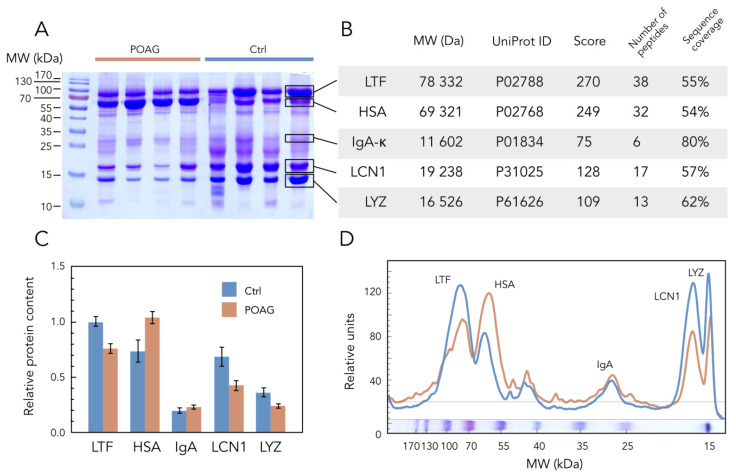
POAG-related alterations in core TF proteins. (**A**) Representative SDS-PAGE image of TF samples obtained from POAG and control (Ctrl) patients (each track contains 20 µL of the sample). Protein standards in kDa are noted in the left column. (**B**) Identification of the protein bands indicated in panel A, using tryptic peptide mass fingerprinting by MALDI-TOF mass spectrometry. Molecular weights (Mr) and accession numbers (UniProt ID) of the proteins and identification scores (Score), as well as the number of detected peptides and sequence coverage data in %, are provided. (**C**,**D**) The average weight fractions of the core tear proteins estimated from the SDS–PAGE of TF samples from POAG and control (Ctrl) groups by densitometric scanning of the bands. Data were handled using GelAnalyzer software version 19.1. Error bars in panel C represent standard deviation (*p* < 0.05 with respect to POAG versus control for all proteins except IgA).

Given that biochemical changes in TF underlying POAG classification affected both major peaks of the denaturation profiles, we hypothesized that they corresponded to the denaturation of the most abundant tear proteins. Indeed, although TF contains up to 1500 different proteins [23], its core proteome is presented by only 5 major components [31], which can measurably contribute to the TFDPs. SDS-PAGE of TF samples obtained in control and POAG groups (Table 1) identified POAG-associated alterations in four core proteins with molecular weights of approximately 15, 20, 70, and 80 kDa, whereas the fifth component (30 kDa) remained unchanged (Figure 2A). Using in-gel trypsin digestion and MALDI-TOF peptide mass fingerprinting (Figure 2B), these proteins were recognized as lysozyme C (LYZ), lipocalin-1 (LCN1), serum albumin (HSA), lactotransferrin (LTF), and immunoglobulin kappa (light chain of IgA; IgA-κ), respectively. All of them were identified as core proteins of normal tear in previous proteomic studies [32,33,34,35,36] and recognized as potential TF biomarkers of POAG [12]. The quantitative analysis of the SDS-PAGE data elucidated a significant increase in HSA content, accompanied by a decrease in concentrations of LYZ, LCN1, and LTF in TF of POAG patients (Figure 2C,D).

### 2.5. Investigation of Denaturation Properties of Tear Using TF-Relevant Protein Mixtures

**Figure 3:**
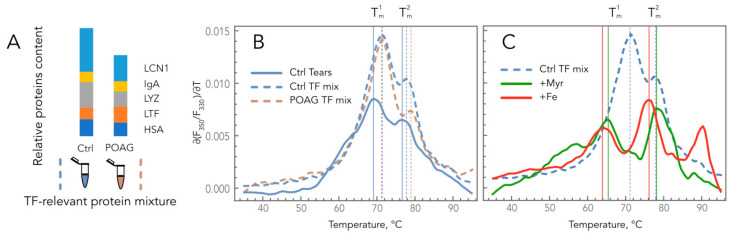
Impacts of the composition and ligand-binding states of the core tear proteins on TFDPs. (**A**) Schematic representation of control and POAG TF-relevant protein mixtures. (**B**) Comparison of denaturation profiles of control and POAG TF-relevant protein mixtures with those of normal TF. (**C**) Effects of myristic acid and iron on denaturation profiles of control TF-relevant protein mixture.

To assess if these alterations contributed to POAG-associated changes in the thermodynamic properties of TF, we compared denaturation profiles of homogenous core tear proteins, as well as their mixtures corresponding to healthy (control) and glaucomatous TF. The experiments were performed at pH 7, corresponding to normal tears [37]. Thermal denaturation of individual LYZ, LCN1, IgA, HSA, and LTF proteins by using Tycho.NT6 instrument was characterized by single transition peaks with maxima at corresponding (∂(F_350_/F_330_)/∂T)T curves at 67.8, 69.4, 72.9, 75.1, and 67.4 °C, respectively. Meanwhile, the denaturation of their mixture presented as a profile with two peaks resembling those of human TF (Figure 3A,B). Moreover, the comparison of control and POAG TF-relevant mixtures revealed a high-temperature shift of T^2^_m_ from 77.7 to 78.9 °C, i.e., similar to those observed in TF of POAG patients. Since most of the examined proteins possess lipid and metal-binding properties, we also analyzed if such ligands would affect the observed profiles. The focus was made on fatty acids (by the example of myristic acid) and iron, as they were previously demonstrated to increase in glaucomatous TF [9,38]. The presence of myristic acid increased the T_m_ of individual HSAs (from 75.1 to 80.3 °C) but decreased it in the case of LYZ (from 67.8 to 55.6 °C), whereas the binding of iron affected only LTF by shifting its melting point towards higher temperatures (from 67.4 to 95.0 °C). Notably, the addition of myristic acid to the control TF-relevant protein mixture yielded a POAG-shaped denaturation profile with a T^1^_m_ and T^2^_m_ of 65.4 and 78.3 °C, respectively (the average values for POAG TF were 64.5 and 79.3 °C) (Figure 3C). The presence of Fe^3+^ also promoted the POAG-like low-temperature shift of T^1^_m_ (from 77.7 to 64.0 °C) and induced the formation of the additional high-temperature peak with a maximum at 90.3 °C. It should be added that the incubation with prostaglandin F2 alpha analog latanoprost (HSA binds prostaglandin F2 alpha [39]), which is commonly used as an IOP-lowering drug in POAG patients, did not produce any effects on the TF-relevant mixtures.

We concluded that the shape and position of TF denaturation profiles are defined by both the composition of the core tear proteins and their ligand-binding states, which change upon POAG development. Therefore, these profiles can be regarded as a complex biomarker of the disease integrating proteomic, lipidomic, and metallomic alterations in TF. Importantly, TFDPs do not appear to depend on the presence of traces of antiglaucoma drugs, thus reflecting only the biochemical alterations associated with the disease.

## 3. Discussion

In this study, we proposed a novel perspective concept of POAG diagnostics based on monitoring biochemical alterations in glaucoma TF by nanoDSF coupled with machine learning. TF represents an informative source of biomarkers, as it can reflect both local and systemic changes associated with glaucoma [14]. Indeed, TF analysis is increasingly considered for diagnostics of diseases that do not directly relate to tear-producing or tear-contacting tissues (lachrymal glands, cornea, sclera, and conjunctiva), such as diabetic retinopathy, cancer, or neurological disorders [40]. The procedure of TF collection is non-invasive and takes only 5 min of a patient’s attendance. The denaturation profiles of TF can be registered using Tycho NT.6 (Nanotemper, München, Germany), originally designed for the quality control of individual proteins. This nanoDSF instrument is clinically friendly since it is compact, easy to manipulate, uses disposable capillaries, requires small amounts of a sample (10 µL), and records the profiles for six samples within only 3 min. Since the subsequent classification of POAG patients is based on machine learning and can be automatized, the overall diagnostic procedure is quite short, which is important for prompt screenings in a clinical setting.

We trialed two approaches to the classification of POAG patients, considering the TFDPs registered by nanoDSF. The first approach involves two-parameter clustering based on the maxima of the first derivative of the thermal dependence of the ratio F_350_/F_330_ (T^1^_m_ and T^2^_m_). According to our in vitro studies, the positions of these maxima are determined by the denaturation of core tear proteins and depend both on their composition and ligand-binding states. Thus, the pathological shifts observed for POAG profiles could reflect alterations in the protein, lipid, and metal content of TF, which are indeed characteristic of the disease (see below). However, these shifts are seen not only in POAG patients but also in about half of the healthy individuals, resulting in a large number of false-positive identifications. Therefore, the cluster analysis based solely on the temperature values of the first and second peaks did not yield the accuracy required for a diagnostic tool that could be utilized in clinical practice. 

The second approach involves machine learning algorithms, which are increasingly used for the automatic recognition of patients with various disorders, such as in diagnostics of cancer based on plasma denaturation profiles, or diagnostics of glaucoma by metabolomic changes in TF [16,27]. Indeed, machine learning is gaining popularity in the analysis of medical-related data for diagnostic purposes [41]. In our case, its application allows for the consideration of changes in profiles based on the complete set of data, without any preprocessing. Due to the substantial individual variability of denaturation curves, these changes in TFDPs of glaucoma patients cannot be detected by other methods, making the machine learning approach the most optimal solution. The employed algorithms consider full-range nanoDSF data, including temperature dependencies of F_330_, F_350_, and F_350_/F_330_ and the respective derivatives. These dependencies seem to be more sensitive to POAG-associated changes in TF composition, as their consideration significantly reduces the number of false-positive identifications. Overall, we suggest that a positive result obtained by the machine-learning-based approach will allow us to reveal individuals with a high probability of POAG who should be subjected to additional examination. Furthermore, the use of both of the above-described approaches may significantly improve the accuracy of prospective glaucoma diagnostics.

The developed classification of the patients is based on POAG-associated alterations predominantly in core TF proteins. A normal tear contains five core proteins, namely LYZ, LTF, LCN1, IgA, and HSA [31]. All of them, except for HSA, are produced by the lachrymal gland and account for up to 85% of the total protein, whereas HSA (together with minor blood-derived proteins) leaks into TF from the conjunctival and other vessels [31,42,43]. Importantly, TF can accumulate components of AH penetrating via scleral percolation in the uveoscleral pathway and thereby become enriched in specific glaucoma-related molecules [14]. The patients from our POAG cohort demonstrated an increase in TF concentration of HSA, accompanied by a decrease in LYZ, LCN1, and LTF. Similar alterations were registered for HSA, LYZ, and LCN1 in previous proteomic studies of glaucomatous tears; however, in the case of LTF, they report an opposite trend [21,22]. It should be emphasized that the alterations in the TF proteome underlying the proposed classifications exhibit no relation to the elderly age of POAG patients (see Table 2). Consistently, the age-dependent changes to human tear composition involve completely different patterns of mainly inflammatory proteins [44].

As was mentioned above, the POAG-type shifts in TFDPs seem to be governed by both the composition and the ligand-binding states of the core tear proteins. In our experiments, individual LYZ, LCN1, IgA, HSA, and LTF demonstrated half-maximal transitions at 67.8, 69.4, 72.9, 75.1, and 67.4 °C, which generally correspond to the previous estimates [45,46,47,48,49]. The increase in the content of “high-melting” HSA compared to “low-melting” LYZ, LCN1, and LTF observed in our POAG patients may contribute to the characteristic high-temperature shift of the second maximum (T^2^_m_) of the TF/TF-relevant profiles. Importantly, LYZ, LCN1, and HSA are capable of binding various lipids [50,51,52,53], which can affect their thermodynamic properties [49,54]. TF has a complex lipid composition, consisting mainly of nonpolar lipids with only ~5% of amphiphilic lipids, including (O-acyl)-o-hydroxy fatty acids, cholesterol, free fatty acids, phospholipids, and ceramides [42,55]. The changes in the TF lipidome accompanying glaucoma remain poorly investigated. Recently, we identified POAG-associated alterations in lipid mediators, such as polyunsaturated fatty acids, oxylipins, and signaling phospholipids [9], but most of them are low-level and hardly affect the properties of TF proteins. In our current experiments, the presence of a fatty acid stabilized HSA (in agreement with previous observations [49]) but significantly reduced the thermal stability of LYZ, which may account for both POAG-type shifts (decrease in T^1^_m_ and increase in T^2^_m_) in TFDPs and TF-relevant profiles. Besides lipids, the core TF proteins can coordinate biometals such as iron (LTF, HSA, and LYZ) or zinc (LCN1, HSA, and LYZ) [56,57,58,59,60]. Notably, an increase in the TF content of iron represents the well-recognized distinctive feature of POAG [38]. According to our observations, LTF undergoes prominent stabilization upon iron binding, and this is in agreement with previous data [46]. In the TF of healthy individuals, the LTF level (25 μM [61]) is approximately 3-fold higher than the iron concentration (9 μM [38]). Thus, most of the tear LTF is present in the Fe^3+^-free state and can function as an iron scavenger, thereby possessing antioxidant activity [62,63]. In the TF of POAG patients, the iron concentration increases 4-fold [38]. Despite being downregulated in glaucomatous tears, LTF can bind this excess iron, leading to the accumulation of its more stable Fe^3+^-bound form. This could be another factor contributing to the high-temperature shift of the second peak (T^2^_m_) in TFDPs and TF-relevant profiles.

Overall, the TFDP can be regarded as a novel integrative biomarker of POAG. Generally, the term “biomarker” can be defined as a “parameter that can objectively be measured and evaluated as an indicator of either normal or pathologic processes, or of a response to a therapeutic intervention” [64]. The core TF proteins fit this definition well, and almost all of them (LYZ, LTF, LCN1, and IgA subunits) have already been proposed as such indicators based on the results of proteomic studies [21,22]. Our approach facilitates their measurement, making it much faster and methodologically easier as compared to mass-spectrometry studies or other approaches proposed earlier. Furthermore, the TFDP integrates not only proteomic but also certain lipidomic and metallomic alterations in TF, thereby increasing the number of molecular components being simultaneously measured in the same probe. The registration of TF denaturation profiles in POAG suspects can be complemented by the analysis of TF biomarkers of the other forms of the disease, such as normal tension glaucoma (BDNF) and primary angle-closure glaucoma (mucin 5AC) [65,66], thereby enabling their differential diagnosis. Further studies are required to trial our approach in larger groups of patients, including those with other frequent ocular diseases, as well as to adapt it for use in a clinical setting.

## 4. Materials and Methods

### 4.1. Materials

The Schirmer tear strips were from Madhu Instruments (New Delhi, India). Myristic acid, human proteins serum albumin (HAS), lactotransferrin (LTF), lysozyme C (LYZ), immunoglobulin A (IgA), and MS-grade trypsin were from Sigma-Aldrich (Saint Louis, MO, USA). Human lipocalin-1 (LCN1) was purchased from MyBioSource (San Diego, CA, USA). Mass spectrometry (MS)-grade trypsin was from Thermo Fisher Scientific, Inc (Waltham, MA, USA). Ferric citrate was from Carlo Erba (Milan, Italy). Other chemicals were from Sigma-Aldrich, Amresco (Solon, OH, USA), or Serva (Heidelberg, Germany) and were at least of reagent grade.

### 4.2. Subjects

TF was collected from 311 ophthalmic patients treated at Helmholtz National Medical Research Center of Eye Diseases (Moscow, Russia). Of these, 171 patients were with refractive anomalies (control subjects), 58 patients with peripheral retinal degeneration (PRD), and 82 patients with POAG. Diagnoses were made by experienced ophthalmologists (SYP, OMF, and NYK). Refractive anomalies were established by autorefractometer (Nidek, Aichi, Japan), under conditions of mydriasis. PRD was detected during fundus examination in mydriasis, using a three-mirror Goldman lens, allowing observation of the retinal periphery. The POAG patient workup was performed in accordance with the National Glaucoma Guidelines [67] and included an IOP measurement (tonometry), visual fields assessment (Heidelberg Edge Perimeter, SAP-II 30-2 program, Heidelberg Engineering, Heidelberg, Germany), and examination of the optic nerve state (cupping, thinning of the neuroretinal rim, notch formation, and disc hemorrhage), using ophthalmoscopy, biomicroscopy, Heidelberg retinal tomography (Heidelberg Retina Tomograph 3, Heidelberg Engineering, Heidelberg, Germany), and optical coherence tomography (Spectralis OCT2, Heidelberg Engineering, Heidelberg, Germany). All studies were conducted in accordance with the Declaration of Helsinki and the Association for Research in Vision and Ophthalmology (ARVO) statement on human subjects and were approved by the local ethical committee of Helmholtz National Medical Research Center of Eye Diseases. The participants signed written informed consent.

### 4.3. TF Collection

TF was collected in patients by using gauged Schirmer’s test paper strips, without anesthesia or tear stimulation. In all subjects, the procedure was performed under identical conditions, namely the same point of time (9–10 a.m.), the fasted state, the same medical personnel, and the same air and light conditions. The strip was placed behind the lower conjunctival sac for 5 min, and the wet fragment was cut off and stored at −70 °C. To extract TF components, the strip was transferred into 25–30 μL of deionized water (1 µL of water per 1 mm of the strip) in a 0.5 mL plastic microcentrifuge tube that was punctured at the bottom by using a G30 needle. The tube was placed in a 1.5 mL microcentrifuge tube, centrifuged at 14,000× *g* for 5 min at 4 °C, and then the supernatant (TF sample) was used for further analysis.

### 4.4. TF Analysis by nanoDSF

Denaturation profiles of the TF samples were obtained using Tycho NT.6 instrument (Nanotemper, München, Germany). Briefly, 10 μL samples were loaded into NT.6 capillaries and heated from 35 °C to 95 °C, at a rate of 0.3 K/s. Six samples were analyzed simultaneously. Raw data were exported into datasets that contained fluorescence intensity at 330 and 350 nm (F_330_ and F_350_), the ratio of these values (F_350_/F_330_), and their first derivatives (∂F_330_/∂T, ∂F_350_/∂T, ∂(F_350_/F_330_)/∂T).

### 4.5. Patients Classification Based on TF Denaturation Profiles

The classification of denaturation profiles was performed using cluster analysis or artificial intelligence (AI) algorithms. Cluster analysis was performed by taking the position of the maxima of two peaks (T^1^_m_ and T^2^_m_) from the first derivative ∂(F_350_/ F_330_)/∂T and applying k-means clustering to the obtained data in Mathematica software version 12.0 (Wolfram Research, Champaign, IL, USA).

AI analysis involved temperature dependences of two nanoDSF outputs (F_330_ and F_350_) and the F_330_/F_350_ ratio, as well as the first derivatives of these functions. The algorithms included Logistic Regression (LR), Support Vector Machine (SVM), and two different ensemble methods, namely Random Forest (RF) and Adaptive Boosting (AdaBoost) [68]. Each algorithm was evaluated using a 5-fold cross-validation approach where the dataset is divided into 5 pieces and the algorithms are trained on 4 and evaluated on the 5th, with each folder being used once on test and belonging to 4 training sets. The resulting accuracies are thus the averages of 5 experiments. Best performances were obtained by using all elements of the nanoDSF data together, and the results reported in this study correspond to this case. The code used was written in Python. The data preparation was carried out using the Pandas library version 1.4.0 (https://pandas.pydata.org), while the machine learning algorithms were run using the Scikit-learn toolbox version 1.1.0 (https://scikit-learn.org). Raw data from the nanoDSF instrument (F_330_ and F_350_) were interpolated using InterpolatedUnivariateSpline from the scipy.interpolate module in order to ensure the same temperature alignment for all data. The different tested implementations were as follows: (1) LogisticRegression from the linear_model module with parameter max_iter sets to 1000; (2) SVC from the svm module with the following combination of parameters—kernel = “poly”, gamma = “auto”, C in (1, 1000) degree between 1 and 3 (best-obtained combination of parameters are kernel = “poly”, degree = 1, C = 1); (3) RandomForestClassifier from the ensemble module with parameter n_estimators fixed to 500; and (4) AdaBoostClassifier from the module ensemble with a DecisionTreeClassifier from the module tree as a weak classifier (parameter base_estimator) with max_depth taken between 1 and 3, n_estimators set to 100 (best results obtained for max_depth = 3). All algorithms were evaluated using the split from the cross_val_score method of the model_selection module with the parameter cv set to 5.

### 4.6. Identification and Quantitative Analysis of TF Proteins

TF proteins were separated by SDS–polyacrylamide gel electrophoresis (SDS-PAGE) and stained with Coomassie brilliant blue R-250. Each SDS-PAGE track was loaded with 20 μL of TF sample. The bands were excised from the gel, washed with 40% acetonitrile and 0.05 M NH_4_HCO_3_, dehydrated in acetonitrile, and then the proteins were digested by trypsin (15 µg/mL in 0.05 M NH_4_HCO_3_) at 37 °C overnight. The peptides were extracted with 0.5% trifluoroacetic acid; mixed on a steel target with 20 mg/mL 2,5-dihydroxybenzoic acid, 20% (*v*/*v*) acetonitrile, and 0.5% (*v*/*v*) trifluoroacetic acid; and analyzed using an ultrafleXtreme MALDI-TOF/TOF mass spectrometer (Bruker Daltonics, Billerica, MA, USA) equipped with a Smartbeam-II laser (Nd:YAG, 355 nm) in reflector mode. Monoisotopic [MH]^+^ molecular ions were detected in the m/z range of 600–5000, with a peptide tolerance of 30 ppm. The mass spectra were handled by flexAnalysis software version 3.3 (Bruker Daltonics), and TF proteins were identified using Mascot software version 2.3.02 (Matrix Science, Boston, MA, USA) and the NCBI protein database.

The weight fractions of core TF proteins were estimated from the SDS–PAGE experiments by densitometric scanning of the bands and data analysis, using GelAnalyzer software version 19.1 (http://gelanalyzer.com/). The data were analyzed with the mean standard deviation method, using SigmaPlot version 11 (SYSTAT Software, San Jose, CA, USA). Statistical significance was evaluated with an unpaired two-tailed *t*-test.

### 4.7. Analysis of TF Proteins and TF-Relevant Protein Mixtures by nanoDSF

Purified TF proteins were dialyzed against 20 mM sodium phosphate buffer (pH 7.0) and examined by nanoDSF individually or as mixtures in physiological TF proportions, corresponding to healthy individuals (140 μg/mL HSA, 110 μg/mL LTF, 50 μg/mL LYZ, 30 μg/mL IgA, and 100 μg/mL LCN1) or POAG patients (110 μg/mL HSA, 150 μg/mL LTF, 30 μg/mL LYZ, 30 μg/mL IgA, and 60 μg/mL LCN1). In some experiments, the protein preparations were analyzed in the presence of 100 μM myristic acid or 1 mM of iron (III) citrate. The procedure of measurement on the nanoDSF instrument was the same as was used for the screening of TF samples (see above).

## Figures and Tables

**Table 1:** Characteristics of experimental groups.

Parameter		Control	POAG	PRD
Number of participants		171	82	58
Mean age ± SD 1, years		31.55 ± 20.07	68.85 ± 10.12	32.05 ± 13.78
Gender (m/f), %		42.10/57.89	37.80/62.20	32.76/67.24
Treatment, %	Beta-blockers	-	56.1	-
	CA inhibitors	-	48.8	-
	Prostaglandin analogs	-	31.7	-
	No treatment		29.3	

**Table 2:** Distribution of patients in groups determined by the different classifying algorithms.

Group	Parameters		K-Means Two-Parameter Clustering		LR (Control versus PRD)		AdaBoost (Control versus POAG)	
			Negative	Positive	Negative	Positive	Negative	Positive
Controls	Patients, %		53.2	46.8	76.0	24.0	86.5	13.5
	Mean age ± SD, years		27.3 ± 9.9	35.6 ± 22.2	31.4 ± 19.8	32.1 ± 20.0	30.0 ± 17.13	47.9 ± 22.1
	Gender (m/f), %		42.8/57.2	33.7/66.3	39.2/60.8	41.5/58.5	40.6/59.4	30.5/69.5
	Accuracy ± CI *, %		-		65.5 ± 2.0		-	
PRD	Patients, %		44.8	55.2	65.5	34.5	-	-
	Mean age ± SD, years		30.4 ± 12.8	33.6 ± 15.7	30.9 ± 14.6	34.3 ± 12.9	-	-
	Gender (m/f), %		50/50	15.7/84.3	26.3/73.7	45.0/55.0	-	-
	Accuracy ± CI *, %		-		-		81.4 ± 2.5	
POAG	Patients, %		28.0	72.0	-	-	30.0	70.0
	Mean age ± SD, years		63.3 ± 12.6	71.0 ± 8.7	-	-	63.2 ± 12.0	71.3 ± 8.15
	Gender (m/f), %		47.8/52.2	33.9/66.1	-	-	35.1/64.9	44.0/56.0
	Treatment, %	β-blockers	52.2	55.9	-	-	52.0	57.9
		CA inhibitors	56.5	44.1	-	-	48.0	49.1
		PG analogs	34.8	28.8	-	-	36.0	29.8
		no treatment	26.0	32.2	-	-	28.0	29.8

* At 95% of the average.

## Data Availability

The data and the code used in this study are available upon request from the corresponding authors.

## Supplementary Materials

The following supporting information can be downloaded at https://www.mdpi.com/article/10.3390/ijms24087132/s1.
