# Supplementary material for: Tear nanoDSF Denaturation Profile Is Predictive of Glaucoma
Source: Int J Mol Sci. 2023 Apr 12;24(8):7132. doi: 10.3390/ijms24087132 (PMC10139145; doi:10.3390/ijms24087132)
Supplement: Supplementary file 1 [file ijms-24-07132-s001.zip › ijms-2319556-supplementary.pdf]

Table S1. Distribution of patients in clusters determined with k-means two-parameter clustering based on TFDP maxima (T^1_m and T^2_m).

Cluster 1

POAG			PRD			control		
number	T^1_m, °C	T^2_m, °C	number	T^1_m, °C	T^2_m, °C	number	T^1_m, °C	T^2_m, °C
G1	63.15	75.45	P1	66.55	77.55	C1	67.95	77.95
G2	68.55	79.85	P2	66.85	79.05	C2	70.55	79.15
G3	67.35	78.75	P3	67.65	79.45	C3	68.25	78.55
G4	68.05	80.05	P4	69.75	78.65	C4	68.25	78.55
G5	67.65	77.95	P5	69.05	78.35	C5	67.45	78.55
G6	67.45	78.45	P6	69.35	78.35	C6	67.15	76.95
G7	69.45	81.25	P7	66.55	77.55	C7	68.05	78.45
G8	66.15	76.65	P8	67.85	77.45	C8	69.05	78.55
G9	69.95	77.85	P9	70.05	77.55	C9	66.75	78.05
G10	66.75	78.45	P10	65.95	76.35	C10	68.05	76.55
G11	68.85	78.35	P11	68.55	76.55	C11	68.85	76.75
G12	69.25	79.35	P12	68.35	77.35	C12	67.55	78.75
G13	68.75	78.05	P13	67.45	75.25	C13	67.75	77.35
G14	68.25	76.95	P14	66.45	77.75	C14	67.15	77.35
G15	68.05	76.65	P15	67.65	78.35	C15	66.25	77.45
G16	70.75	79.95	P16	71.55	75.45	C16	66.25	75.25
G17	68.25	79.15	P17	69.55	75.35	C17	71.35	79.05
G18	68.75	79.55	P18	68.75	75.45	C18	66.35	75.55
G19	67.15	77.45	P19	67.25	77.75	C19	68.95	73.65
G20	70.85	79.55	P20	70.15	75.65	C20	68.95	77.95
G21	70.45	79.55	P21	67.85	78.45	C21	65.95	77.95
G22	65.85	77.55	P22	67.35	76.95	C22	67.05	78.65
G23	69.05	77.35	P23	66.05	76.85	C23	67.25	78.45
			P24	67.55	75.65	C24	68.45	78.55
			P25	67.05	78.35	C25	66.75	78.75
			P26	68.65	76.95	C26	68.35	74.95
						C27	66.45	78.55
						C28	66.75	76.85
						C29	68.35	76.85
						C30	68.35	76.05
						C31	67.65	79.55
						C32	68.95	78.15
						C33	68.75	77.95
						C34	66.65	78.35
						C35	67.35	77.15
						C36	68.15	77.75
						C37	68.05	78.05
						C38	67.25	78.25
						C39	69.15	78.95
						C40	69.15	78.95
						C41	67.45	78.55
						C42	68.15	79.25
						C43	68.65	76.95
						C44	69.25	76.85
						C45	65.75	76.65

						C46	65.95	76.95
						C47	69.85	77.95
						C48	68.45	77.45
						C49	69.35	78.65
						C50	68.15	78.55
						C51	69.65	79.35
						C52	69.35	79.05
						C53	69.05	79.05
						C54	67.05	78.35
						C55	67.65	75.35
						C56	63.75	76.45
						C57	65.45	77.85
						C58	66.85	78.55
						C59	68.35	76.85
						C60	68.45	76.85
						C61	70.55	77.45
						C62	61.15	71.95
						C63	65.85	76.25
						C64	67.05	76.65
						C65	68.75	78.05
						C66	67.95	77.45
						C67	68.55	77.55
						C68	69.65	78.05
						C69	67.55	76.95
						C70	67.65	77.45
						C71	67.55	78.55
						C72	68.35	77.75
						C73	67.95	78.55
						C74	67.75	78.45
						C75	66.55	78.25
						C76	68.15	77.45
						C77	67.95	78.95
						C78	67.15	77.15
						C79	68.55	77.35
						C80	67.75	77.35
						C81	66.95	76.65
						C82	67.35	77.35
						C83	67.25	78.35
						C84	69.45	79.55
						C85	67.25	77.65
						C86	67.55	77.75
						C87	68.75	77.45
						C88	67.65	78.05
						C89	68.05	78.65
						C90	67.05	77.35
						C91	68.85	77.75

Cluster 2

POAG			PRD			control		
number	T^1_{m}, °C	T^2_{m}, °C	number	T^1_{m}, °C	T^2_{m}, °C	number	T^1_{m}, °C	T^2_{m}, °C
G24	63.15	80.45	P27	67.35	80.85	C92	63.55	79.75
G25	64.1	81.3	P28	67.35	80.05	C93	67.95	81.55
G26	64.05	79.05	P29	63.65	78.75	C94	63.55	79.75
G27	63.75	81.75	P30	65.45	79.15	C95	66.95	80.45
G28	64.25	77.95	P31	67.45	79.95	C96	64.35	80.95
G29	63.15	81.15	P32	65.75	79.55	C97	66.65	80.85
G30	63.05	76.75	P33	67.85	80.05	C98	68.05	83.05
G31	63.25	78.65	P34	62.75	77.85	C99	64.35	78.55
G32	60.65	78.15	P35	65.45	78.95	C100	66.75	80.45
G33	65.35	80.75	P36	63.25	78.25	C101	66.55	78.95
G34	52.45	74.25	P37	62.45	77.15	C102	63.55	79.75
G35	66.25	78.75	P38	61.65	78.95	C103	67.15	79.55
G36	66.25	81.55	P39	64.45	78.85	C104	66.15	79.35
G37	65.95	79.75	P40	62.95	77.45	C105	64.85	78.15
G38	63.35	80.25	P41	68.25	80.95	C106	64.95	79.75
G39	64.05	79.85	P42	62.05	78.55	C107	64.75	79.95
G40	56.85	77.25	P43	55.15	77.05	C108	65.35	79.45
G41	66.65	80.05	P44	64.05	79.65	C109	62.95	80.45
G42	66.05	81.25	P45	65.05	79.15	C110	64.55	78.65
G43	64.55	78.15	P46	65.15	78.95	C111	64.95	79.05
G44	64.05	78.15	P47	65.15	78.65	C112	65.65	80.05
G45	64.45	79.55	P48	63.05	76.65	C113	65.05	79.85
G46	66.65	80.05	P49	61.95	76.95	C114	65.75	80.85
G47	64.85	80.55	P50	65.85	79.85	C115	65.25	78.45
G48	63.85	79.15	P51	62.05	76.75	C116	62.15	78.35
G49	64.85	80.55	P52	67.05	80.05	C117	64.35	81.05
G50	66.25	79.75	P53	65.55	79.55	C118	66.65	79.55
G51	65.05	80.55	P54	62.25	79.65	C119	65.35	80.15
G52	63.85	79.65	P55	61.25	79.35	C120	61.85	77.95
G53	63.05	78.15	P56	61.55	76.85	C121	65.45	79.85
G54	65.55	79.65	P57	63.95	77.25	C122	66.15	78.65
G55	64.35	79.65	P58	65.55	78.05	C123	63.75	78.95
G56	65.75	81.25				C124	65.75	79.05
G57	65.75	79.65				C125	66.45	79.15
G58	63.55	79.75				C126	64.05	77.35
G59	65.95	79.95				C127	64.55	78.75
G60	65.45	79.95				C128	64.65	79.35
G61	63.55	81.35				C129	63.15	77.45
G62	64.15	79.75				C130	63.05	78.35
G63	65.65	79.85				C131	66.25	79.25
G64	64.25	79.15				C132	66.95	79.55
G65	63.95	79.35				C133	62.35	79.35
G66	66.35	80.05				C134	62.45	78.75
G67	65.65	79.95				C135	66.95	80.45
G68	66.65	79.65				C136	65.35	79.25
G69	65.25	79.65				C137	65.95	78.95
G70	65.75	80.35				C138	64.65	78.55

G71	65.75	79.85				C139	65.55	79.35
G72	63.85	78.35				C140	65.55	79.85
G73	63.15	79.75				C141	65.25	79.25
G74	64.35	80.15				C142	63.55	78.65
G75	66.65	80.85				C143	62.55	80.25
G76	65.05	80.15				C144	65.45	80.35
G77	64.15	79.75				C145	62.85	80.75
G78	65.1	81.7				C146	66.55	79.65
G79	64.35	79.45				C147	64.45	77.45
G80	64.15	77.85				C148	66.95	79.25
G81	64.85	77.65				C149	63.65	78.55
G82	65.25	78.55				C150	64.65	78.65
						C151	63.55	81.75
						C152	65.65	78.25
						C153	64.65	79.55
						C154	64.65	79.35
						C155	64.25	78.35
						C156	68.35	80.35
						C157	64.35	78.95
						C158	64.05	78.75
						C159	64.85	79.95
						C160	63.95	78.15
						C161	62.15	78.65
						C162	63.45	78.35
						C163	66.35	79.75
						C164	66.55	79.65
						C165	64.65	78.95
						C166	64.05	78.15
						C167	65.95	78.85
						C168	64.45	77.95
						C169	64.65	78.05
						C170	64.85	77.95
						C171	64.25	78.85
